# The Superior Parietal Lobule of Macaque Monkey: Relative Influence of Gaze and Static Arm Position during Reaching

**DOI:** 10.1523/ENEURO.0362-21.2021

**Published:** 2022-01-05

**Authors:** Marina De Vitis, Marta Tabanelli, Rossella Breveglieri, Matteo Filippini, Claudio Galletti, Patrizia Fattori

**Affiliations:** 1 Department of Biomedical and Neuromotor Sciences, University of Bologna, 40126 Bologna, Italy; 2 Alma Mater Research Institute for Human-Centered Artificial Intelligence (Alma Human AI), University of Bologna, 40121 Bologna, Italy

**Keywords:** gaze, posterior parietal cortex, proprioception, reaching movements, superior parietal lobule, visuomotor integration

## Abstract

The superior parietal lobule (SPL) integrates somatosensory, motor, and visual signals to dynamically control arm movements. During reaching, visual and gaze signals are used to guide the hand to the desired target location, while proprioceptive signals allow to correct arm trajectory, and keep the limb in the final position at the end of the movement. Three SPL areas are particularly involved in this process: V6A, PEc, PE. Here, we evaluated the influence of eye and arm position on single neuron activity of these areas during the holding period at the end of arm reaching movements, when the arm is motionless and gaze and hand positions are aligned. Two male macaques (*Macaca fascicularis*) performed a foveal reaching task while single unit activity was recorded from areas V6A, PEc, and PE. We found that at the end of reaching movements the neurons of all these areas were modulated by both eye position and static position of the arm. V6A and PEc showed a prevalent combination of gaze and proprioceptive input, while PE seemed to encode these signals more independently. Our results demonstrate that all these SPL areas combine gaze and proprioceptive input to provide an accurate monitoring of arm movements.

## Significance Statement

This study shows that the SPL areas V6A, PEc, and PE combine eye and static arm positions signals to build an estimate of the limb posture at the end of a reaching movement. The degree of integration of gaze and proprioceptive information changes from a joint processing of these signals in the caudal-most areas V6A and PEc (Brodmann area 7), to a more independent encoding in PE (Brodmann area 5). Our results support the existence of a functional trend in the SPL, with the anterior part dealing mainly with limb representation based on proprioception and the posterior one linking gaze and arm position signals for encoding reaching.

## Introduction

**Figure 1.**
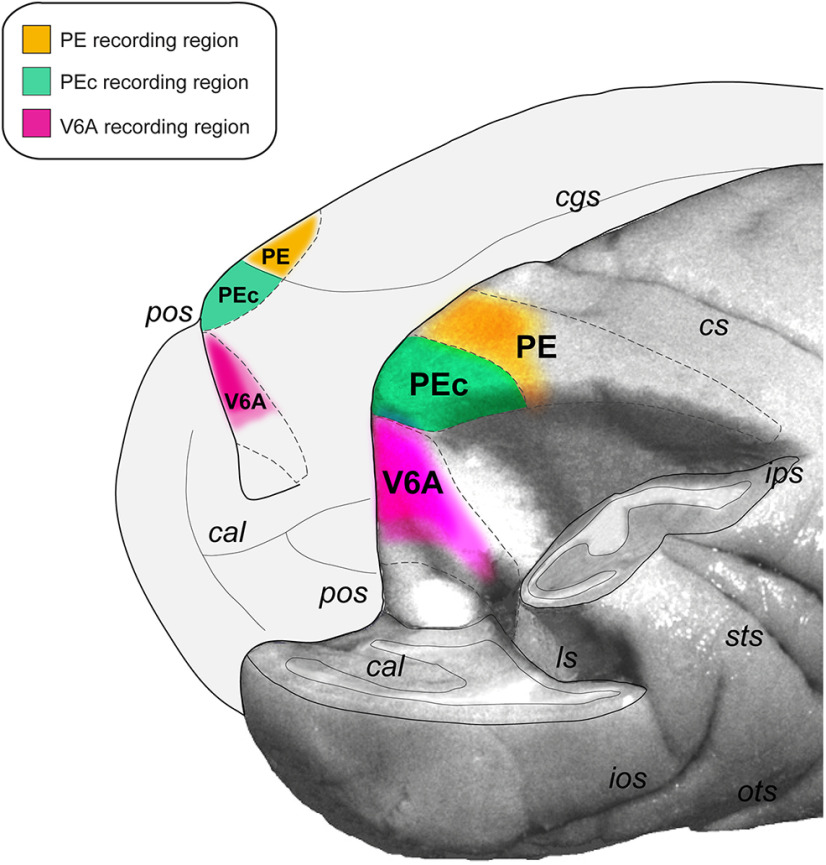
Areas of the superior parietal lobule in a macaque brain. Posterolateral view of a part of *M. fascicularis* brain showing location and extent of areas V6A, PEc, and PE (outlined by gray dashed lines) of the SPL. The right hemisphere is partially dissected to show the areas hidden in the parieto-occipital and intraparietal sulci. Colored areas represent the reconstructions of the recording regions within these areas as a mean of two animals and four hemispheres. cal, calcarine sulcus; cgs, cingulate sulcus; cs, central sulcus; ios, inferior occipital sulcus; ips, intraparietal sulcus; ls, lunate sulcus; ots, occipitotemporal sulcus; pos, parieto-occipital sulcus; sts, superior temporal sulcus (modified from Gamberini et al., 2020).

Eye-hand coordination is a basic function that allows primates to interact with the surrounding environment. In goal directed arm movements, we first direct the gaze to the target, then move the limb toward it and, at the end of movement, hold the arm in the final desired position to allow target touching, pushing, grasping, or manipulation. The basis of these abilities is the integration of eye position with proprioceptive signals from the limbs, an operation that typically occurs in the superior parietal lobule (SPL; Battaglia-Mayer et al., 2001; McGuire and Sabes, 2011; Granek et al., 2012; Fattori et al., 2017). The SPL areas V6A, PEc, and PE (Fig. 1; Pandya and Seltzer, 1982; Galletti et al., 1999) are particularly involved in this process (Galletti and Fattori, 2003; Gamberini et al., 2011, 2018, 2020; Fattori et al., 2017). PE is an area rich in somatosensory cells which receives proprioceptive input from the limbs and is involved in the monitoring of limb posture and movement. It contains mainly neurons sensitive to joint rotations, activated by passive movements of the arm, but also cells spatially tuned by active reaching movements, suggesting that PE is mainly involved in the somatosensory monitoring of arm state during reaching (Duffy and Burchfiel, 1971; Sakata et al., 1973; Mountcastle et al., 1975; Georgopoulos et al., 1984; Kalaska et al., 1990; Kalaska, 1996; Gardner et al., 2007; De Vitis et al., 2019). PEc hosts somatosensory and visual neurons, as well as bimodal (somato-visual) cells modulated by arm movement and visual stimulation, and cells modulated by the direction of gaze. PEc is suggested to use these signals to perform a visuo-somatomotor control of reaching (Battaglia-Mayer et al., 2001; Ferraina et al., 2001; Breveglieri et al., 2006, 2008; Hadjidimitrakis et al., 2015; Piserchia et al., 2017; Gamberini et al., 2018). V6A is a visuomotor area that hosts neurons with sensory features comparable to those of PEc, but with a higher proportion of visual neurons (Gamberini et al., 2011, 2018) and of cells modulated by the direction of gaze (Galletti et al., 1995), but less somatosensory cells than PEc (Breveglieri et al., 2002). Therefore, in both PEc and V6A, somatosensory and gaze stimuli are used for reaching control but the degree of independent encoding of these signals in the two areas is still under debate.

In this article, we have investigated how arm-related and gaze-related signals influence single neurons of these areas by looking at the activity of these neurons at the end of a Fixation-to-Reach task, when both gaze and arm were stationary in space and time. The results showed that V6A, PEc, and PE neurons were differently modulated by gaze and arm-related signals. Area PE was more sensitive to limb proprioceptive input while PEc and V6A were more sensitive to proprioceptive and gaze signals, with the latter particularly modulated by the interaction between gaze and hand position.

## Materials and Methods

### Experimental procedures

Two male macaque monkeys (*Macaca fascicularis*), weighing 4 and 4.6 kg, were involved in this study. The experiments were performed in accordance with the guidelines of EU Directives (86/609/EEC; 2010/63/EU) and Italian national laws (D.L.116–92, D.L. 26–2014) on the protection of animals used for scientific purposes. Protocols were approved by the Animal-Welfare Body and from the Italian Ministry of Health. During training and recording sessions, particular attention was paid to any behavioral and clinical signs of pain or distress.

### The Fixation-to-Reach task

**Figure 2.**
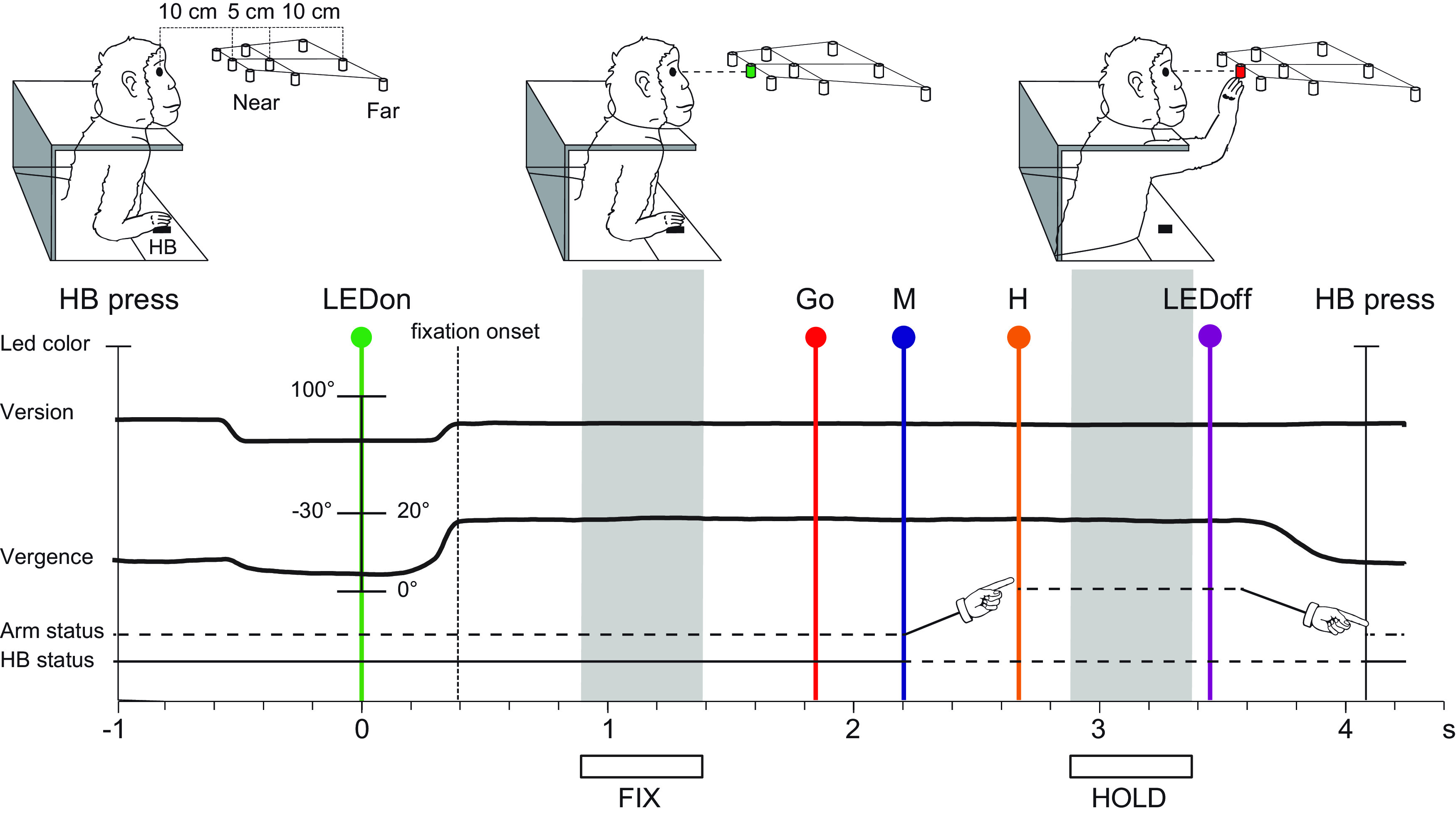
Scheme of the experimental setup and timing of the task. The monkey sat in a primate chair in front of a horizontal panel located at eye level with nine LEDs used both as fixation and reaching targets. HB, home button. The distances in depth between the three targets of the central row from mid-eye level are shown. The time sequence of task events shows LED status, the eye’s vergence and version traces, arm status, and HB status. From left to right, vertical lines indicate, respectively, trial start (HB press, black line), target appearance (LEDon, green line), fixation onset (end of saccade movement, dashed line), go signal (Go, red line), arm movement onset (M, blue line), holding phase of the target (H, orange line), turning off of the LED (LEDoff, purple line), and trial end (HB press, gray line). Arm drawings indicate the forward and backward arm movement. The relevant time intervals (epochs) used for the analysis of neural activity are indicated with gray areas and white bars below the time axis: FIX = fixation epoch, HOLD = holding epoch.

The animal sat in a primate chair (Crist Instruments) and performed a Fixation-to-Reach task. During this task, the monkey sat in front of a horizontal panel located at eye level with nine light-emitting diodes (LEDs; 6 mm in diameter) placed at different distances and directions used as fixation and reaching targets (Fig. 2). Since the targets were aligned at eye level, they could potentially obscure each other. We got the problem solved by gradually masking the LEDs, going from the thinner nearest targets to the ticker farthest one. Thus, the monkeys were able to easily discriminate them. The task was performed in darkness with the hand contralateral to the recording site. In the starting position, the monkey kept its hand on a button [hereafter called the home button (HB), 2.5 cm in diameter] placed 4 cm in front of the chest, outside the animal’s field of view (Fig. 2). Target LEDs were arranged in three rows: one central, along the sagittal midline, and two laterals, at version angles of −15° and +15°, respectively. Along each row, three LEDs were located at different depth, at vergence angles of 17.1°, 11.4°, 6.9°. The nearest targets were located at 10 cm from the eyes, whereas the LEDs placed at intermediate and far positions were located at 15 and 25 cm, respectively (Fig. 2). Target positions were chosen to be all within the peripersonal space.

A trial began when the monkey pressed the HB (Fig. 2, HB press). After 1 s, one of the nine LEDs was switched on to green and the monkey had to fixate the LED while keeping the HB button pressed (Fig. 2, LEDon). Then, the monkey had to wait 1.5–2.5 s for a change in the color of the same LED (from green to red) without performing any eye or arm movement (Fig. 2, epoch FIX). The color change was the go signal for the animal to release the HB and start an arm movement toward the target. Once reached the target, the animal was required to hold the hand on it for 0.8–1.2 s (Fig. 2, epoch HOLD). Target switching off cued the monkey to release it and return to the HB, which ended the trial and allowed the monkey to receive its reward. Note that during FIX the monkey maintained the gaze still on one of the nine LEDs while the arm was located near the body because the hand was pressing the HB; during HOLD, the animal maintained fixation on the target LED while pushing it, so the arm was extended and motionless, and the hand was far from the body.

Stimuli presentation and animals’ performance were monitored using custom software written in Labview (National Instruments), as described previously (Kutz et al., 2005); if monkey broke fixation, made an incorrect arm movement, or did not respect the temporal constraints of the task, the trial was aborted. Microswitches (monopolar microswitches, RS Components) were mounted under the HB and under each LED to monitor the correct performance of arm movements. Eye position signals were sampled with two cameras (one for each eye) of an infrared eye-tracking system (ISCAN) at 100 Hz and were controlled by an electronic window (4° × 4°) centered on the fixation target. If the monkey fixated outside this window, the trial was aborted. The task was performed in darkness, in blocks of 90 randomized trials, 10 for each LED target position.

At the beginning of each recording session, the monkey was required to perform a calibration task to calibrate the eye tracker. In this task, animal fixated 10 LEDs mounted on a frontal panel at 15 cm from the eyes. For each eye, signals to be used for calibration were extracted during the fixation of five LEDs, one central aligned with the eye’s straight-ahead position, and four peripheral ones placed at an angle of ±15° (distance: 4 cm) both in the horizontal and vertical axes. From the two individual calibrated eye position signals, we derived the mean of the two eyes (the conjugate or version signal), and the difference between the two eyes (the disconjugate or vergence signal) using the equations: Version = (R + L)/2 and Vergence = L − R, where R and L were the position of the right and left eye, respectively, expressed in degrees. The version and vergence values were also used by the LabVIEW software to control the gaze position and abort trials in case of incorrectness.

### Surgical and recording procedures

After training completion, a head-restraint system and a recording chamber were surgically implanted in asepsis and under general anesthesia (sodium thiopental, 8 mg/kg/h, i.v.) following the procedures reported in Galletti et al. (1995). Adequate measures were taken to minimize pain or discomfort. A full program of postoperative analgesia (ketorolac trometazyn, 1 mg/kg, i.m., immediately after surgery, and 1.6 mg/kg, i.m., on the following days) and antibiotic care [Ritardomicina® (benzathine benzylpenicillin + dihydrostreptomycin + streptomycin) 1–1.5 ml/10 kg every 5–6 d] followed the surgery.

Single-cell activity was extracellularly recorded from areas V6A, PEc, and PE of the two monkeys (Fig. 1). We performed single microelectrode penetrations using a 5-channel multielectrode recording system (MiniMatrix, Thomas Recording, GmbH). The electrode signals were amplified (at a gain of 10,000) and bandpass filtered (between 0.5 and 5 kHz). Action potentials in each channel were isolated online with a waveform discriminator (Multi Spike Detector; Alpha Omega Engineering). Spikes were sampled at 100 kHz. The present study includes neurons assigned to areas V6A, PEc, and PE following the cytoarchitectonic criteria of Pandya and Seltzer (1982) and Luppino et al. (2005).

### Data analysis

All the analyses were performed using custom scripts in MATLAB (MathWorks, RRID: SCR_001622). Analysis of the neuronal activity during the Fixation-to-Reach task was made by quantifying the discharge recorded during each trial in the following time epochs (Fig. 2):

- FIX: from 500 ms after fixation onset (corresponding to the onset of ocular fixation inside the electronic window) until 1000 ms after it. It contains the neural discharge for LED fixation, avoiding transient saccade-related responses (see Kutz et al., 2003).
- HOLD: from 200 ms after LED pressing until 700 ms after it. It contains the discharge of the cells during hand holding, avoiding transient responses related to the stop of the arm movement.

We included in the analyses only those units recorded during at least seven trials per spatial position, and with a mean firing rate in HOLD and/or in FIX higher than three spikes/s in at least one position. The reasons for these conservative criteria are dictated by the intrinsic high variability of biological responses in the PPC as explained in detail in Kutz et al. (2003).

To assess the effect of the eye and arm position on V6A, PEc, and PE cells, we performed a two-way ANOVA with factors being the epoch (two levels: FIX and HOLD) and target positions (nine levels: nine spatial positions of the reaching targets). FIX was chosen as a reference because in this epoch the gaze was still and the monkeys were not required to execute any arm movement. We defined as task-related and further analyzed those cells showing significant main effects of both target positions and epoch (*p* < 0.05), significant interaction (target positions*epoch, *p* < 0.05), or a cumulative main and interaction effect (epoch+interaction, target positions+interaction).

Significant modulation of neural activity by the target position in each epoch of interest was assessed by a one-way ANOVA (factor: target position, *p* < 0.05). The incidence of task-related cells with significant modulations by the target position during HOLD, FIX and both FIX&HOLD epochs was compared in the three areas V6A, PEc, PE with a z-test (Zar, 1999), as detailed in Fluet et al. (2010). To perform this test, the SE of the sampling distribution difference between two proportions was computed as:

$$SE=\sqrt{p\left(1-p\right)[(1/n1)(1/n2)]}$$

with *p* = [(*n*1 × *p*1)(*n*2 × *p*2)]/(*n*1 + *n*2) representing the pooled sample proportion and *n*1/*p*1 and *n*2/*p*2 representing the size and proportion, respectively, of each sample. Subsequently, the *z score* was calculated as *z* = (*p*1 – *p*2)/SE, and its corresponding *p* value was obtained from the (cumulative) normal distribution.

A z-test was also used to compare the incidence of task-related cells with higher firing rate during HOLD with respect to FIX in all the nine positions (referred as excited cells) and with lower firing rate during HOLD with respect to FIX in all the nine positions (referred as inhibited cells).

To analyze the spatial tuning of task-related cells activity during the time course of the task, a stepwise multiple linear regression model was applied with a sliding window approach (window-bin width: 250 ms; step: 50 ms). A similar method has been used in previous publications from our lab (Hadjidimitrakis et al., 2014a, 2015; De Vitis et al., 2019). To dynamically relate the neural activity to the different target positions over time, we applied the following equation for the firing rate using this regression model:

A(Xi,Yi)=b0+b1Xi+b2Yi

where A was the neural activity in spikes per second for the ith trials; Xi and Yi the positions of the target defined as vergence and version angles, respectively, of the eyes; b1 and b2 were regression coefficients and b0 the intercept. After being tested for their significance, the vergence and version coefficients were normalized with the standard deviation of vergence and version, correspondingly. In each bin, the sign of the significant linear correlation coefficients was used to determine the spatial preference per each neuron.

Population responses of neurons modulated by the target position during HOLD and FIX&HOLD epochs were computed as averaged spike density functions (SDFs). An SDF was calculated (Gaussian kernel, half-width 40 ms) for each neuron included in the analysis and averaged across all the trials for each target position. The peak discharge of the neuron found over all the nine target positions during the epoch of interest (HOLD or FIX) was used to normalize all the SDFs. The normalized SDFs were then averaged to obtain population responses (Marzocchi et al., 2008). To statistically compare the population SDFs curves of best and worst positions in each area, we performed a permutation test (10,000 iterations), comparing the sum of squared errors of the actual and randomly permuted data. Comparisons of responses to target fixation have been made in the interval from 500 to 1000 ms after saccade offset for FIX. Comparisons of responses related to static positions of the arm have been made in the interval from 200 ms after the LED pressing until 700 ms after it for HOLD. The onset of spatial selectivity was calculated as the time of divergence of population SDFs of the best and worst target position (half-Gaussian kernel, width 5 ms).

## Results

We have investigated the influence of gaze and proprioceptive signals from the arm on the activity of neurons of three SPL areas (V6A, PEc, PE) in two macaque monkeys. Animals performed a Fixation-to-Reach task being instructed to fixate and reach nine foveated targets located at different spatial locations in the 3D space facing the animal (Fig. 2). Only the horizontal plane at eye level was explored to reduce the factors influencing neuronal activity, being well known that gaze elevation modulates the activity of neurons in the caudal part of SPL (Galletti et al., 1995; Breveglieri et al., 2012). The task allowed us to test the influence of gazing different positions of the peripersonal space (epoch FIX) and of holding the arm in different spatial configurations (epoch HOLD) on neuronal activity.

We recorded the activity of 303 single V6A cells (left hemisphere: 218, right hemisphere: 85; Monkey A: 168, Monkey B: 135), 264 PEc cells (left hemisphere: 159, right hemisphere: 105; Monkey A: 157, Monkey B: 107), and 189 PE cells (left hemisphere: 91, right hemisphere: 98; Monkey A: 69, Monkey B: 120). We analyzed neural responses during two epochs: target fixation (FIX, from 500 ms after fixation onset until 1000 ms after it; Fig. 2) and target holding (HOLD, from 200 ms after the LED pressing until 700 ms after it; Fig. 2).

### Effect of eye and arm position signals on V6A, PEc, and PE

We examined how many V6A, PEc, and PE neurons were significantly modulated by the eye and arm position (two-way ANOVA, *p* < 0.05) and identified them as “task-related”. A total of 226/303 V6A cells (75%), 188/264 PEc cells (71%), and 85/189 PE cells (45%) showed task-related activity and were further analyzed.

**Figure 3.**
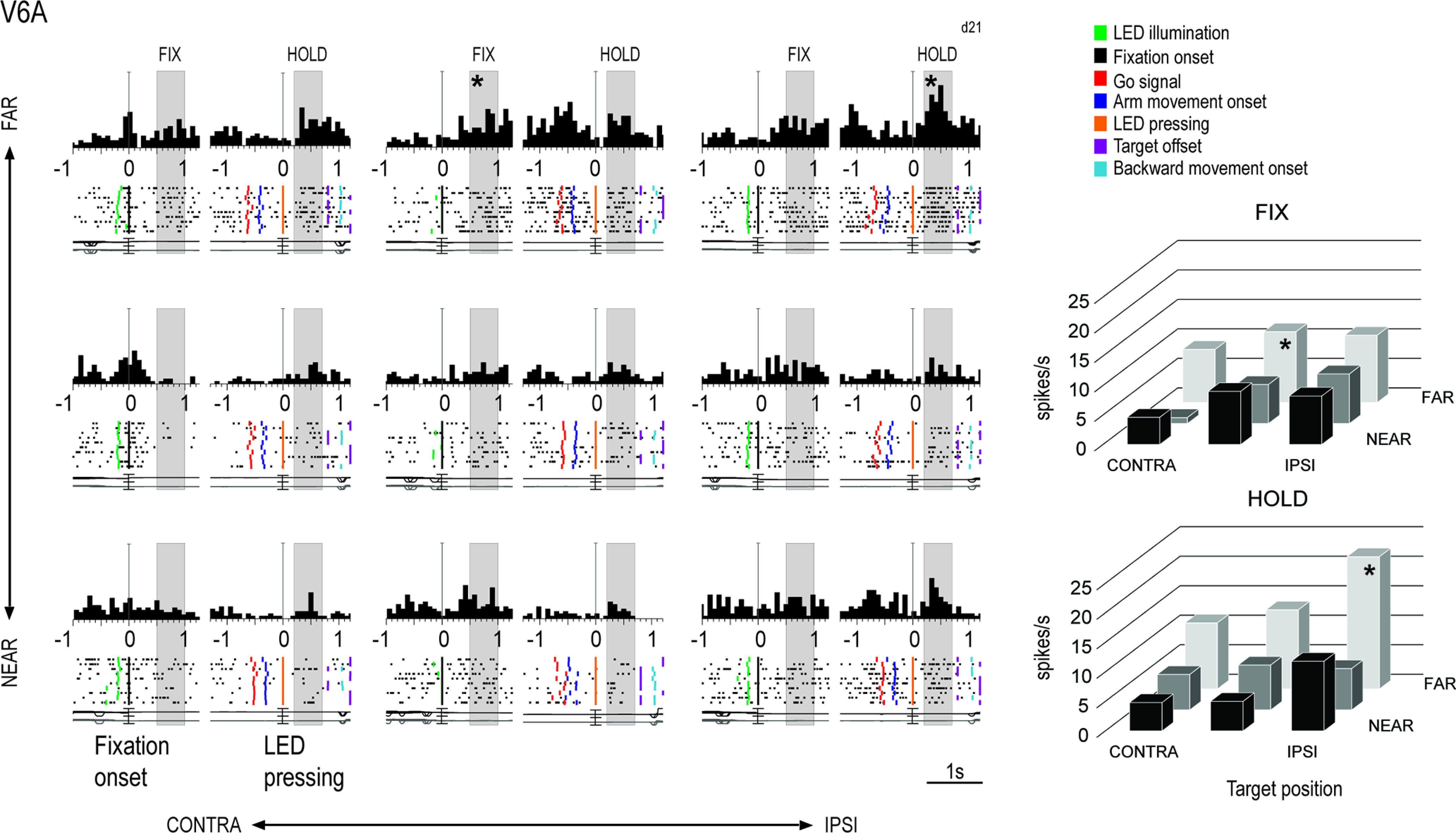
Example V6A neuron tuned by eye/arm positions both in FIX and HOLD. Left, Spike histograms (top), rasters (middle), eye traces (bottom) are shown for each of the nine target positions tested. Target positions were arranged in three directions (columns; contralateral, central, ipsilateral with respect to the recording hemisphere) and three depths (rows; far, intermediate, near with respect to the monkey’s body). Colored vertical lines along rasters indicate behavioral markers that, from left to right, are: LED illumination, fixation onset, go signal, movement onset, movement end (LED pressing), target offset, backward movement onset. Thin vertical lines along spike histograms indicate the alignment of activity at the fixation onset and LED pressing, respectively. Realignment is evidenced with a gap in histograms. Epochs of interest are represented within gray rectangles. Vertical scale on histograms: 40 spikes/s. Right, Distribution of the mean activity of the same cell across trials during epochs FIX (top) and HOLD (bottom) for each of the nine target positions tested. Asterisks indicate the spatial position evoking the highest discharge in each epoch.

**Figure 4.**
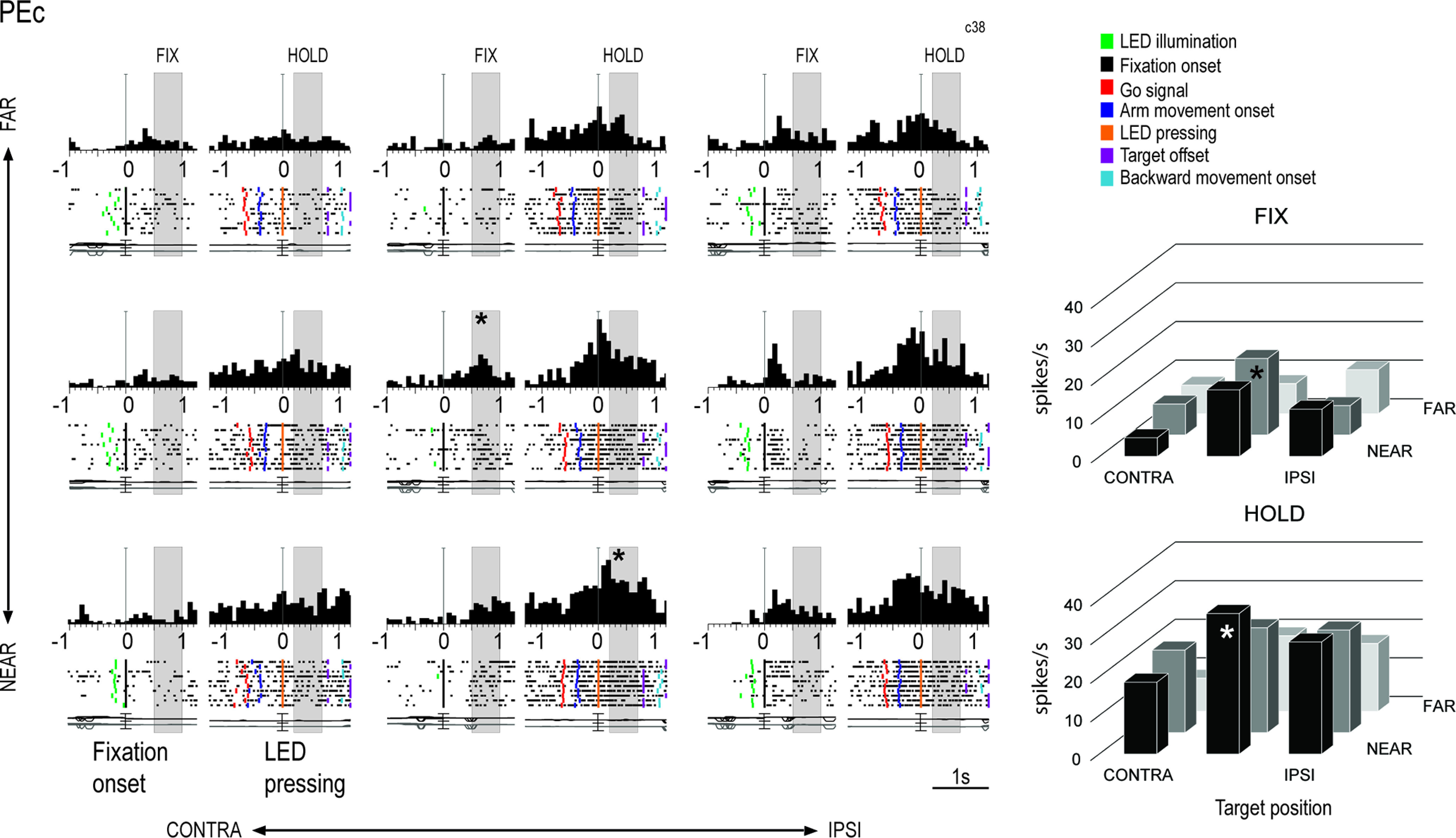
Example PEc neuron tuned by eye/arm positions both in FIX and HOLD. All the conventions as in Figure 3. Vertical scale on histograms: 70 spikes/s.

**Figure 5.**
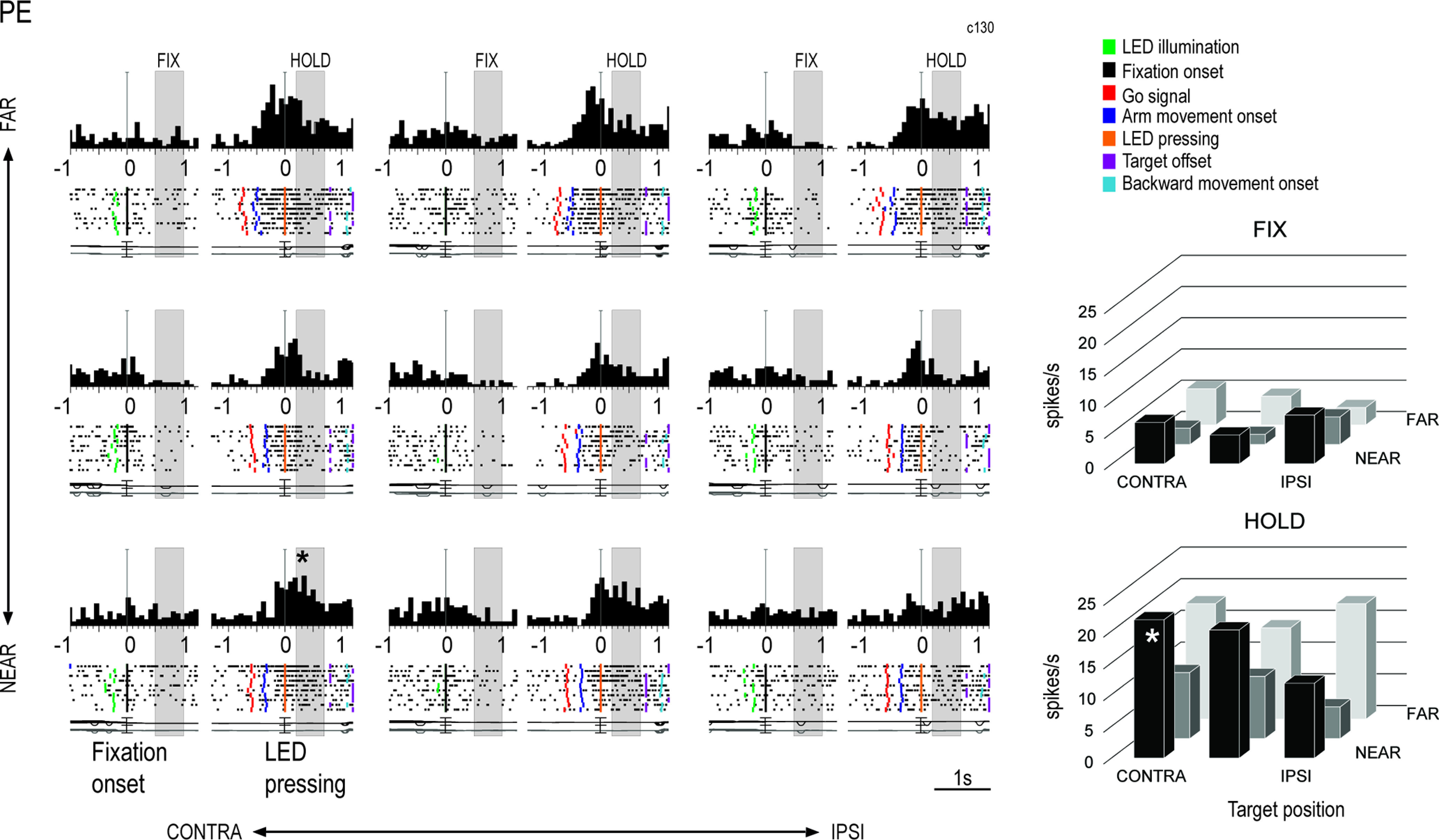
Example PE neuron tuned by eye/arm positions only in HOLD. All the conventions as in Figure 3. Vertical scale on histograms: 48 spikes/s.

Figures 3-5 show three examples of neurons modulated during the Fixation-to-Reach task, recorded from the areas V6A, PEc, and PE, respectively. Task-related V6A neuron showed in Figure 3 was modulated by the spatial position of reaching target both in FIX (one-way ANOVA, *p* = 3 × 10^−5^) and HOLD (one-way ANOVA, *p* = 3 × 10^−8^) and showed a higher discharge during HOLD for far targets, mainly the ipsilateral one. This cell displayed low activity during arm movement and peaked in discharge after the target LED pressing, when the monkey’s arm was still and extended (HOLD). Task-related PEc neuron showed in Figure 4 exhibited a spatial preference for positions ipsilateral and near the body (one-way ANOVA, *p* = 0.001 during FIX, *p* = 10^−6^ during HOLD). Cell’s activity gradually increased after the go signal, peaked around the target LED pressing and decreased afterward, but remained quite high during the HOLD epoch. In contrast to V6A, PE neuron discharged strongly during arm movement (Fig. 5). Its activity was modulated by the spatial position of the arm during HOLD (one-way ANOVA, *p* = 5 × 10^−5^), whereas the activity during FIX was comparable in the nine target positions (*p* = 0.05).

**Figure 6.**
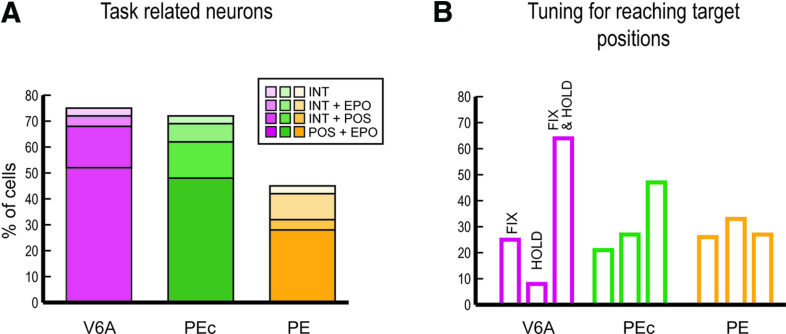
Significant effects modulating V6A, PEc, and PE cells. ***A***, The histograms show the results of a two-way ANOVA as the incidence of cells modulated by the fixation and arm holding in space (EPO: epoch) and the nine spatial positions in one or both the epochs of interest (POS: target positions), or by the interaction of the two factors (INT: target positions*epoch). Numbers of modulated cells for each subgroup of task-related cells: POS+EPO, V6A *N* = 157, PEc = 126, PE = 52; INT+EPO, V6A *N* = 12, PEc = 18, PE = 19; INT+POS, V6A *N* = 47, PEc = 36, PE = 8; INT, V6A *N* = 10, PEc = 8, PE = 6 (cells with no effect, V6A *N* = 27, PEc = 22, PE = 36 not shown in figure). ***B***, Percentages of cells tuned by the position of the reaching target in FIX, HOLD, and FIX&HOLD, as a result of a one-way ANOVA.

Different functional features were observed in PE on one side and in PEc/V6A on the other: firstly, a lower incidence of task-related cells was found in PE compared with both PEc and V6A (z-test, PE vs PEc *p* = 2 × 10^−8^, PE vs V6A *p* = 5 × 10^−6^), whereas no significant difference was found between V6A and PEc (z-test, *p* = 0.4). We categorized neurons in four classes, according to their modulation by fixation and arm holding in space (epoch, EPO in Fig. 6*A*) and by the nine spatial positions (target positions, POS in Fig. 6*A*) during one or both the epochs of interest. Therefore, we distinguished: (1) neurons significantly modulated by the eye position, the arm position in space, or by both (POS+EPO; Fig. 6*A*); (2) neurons modulated only by the interaction POS*EPO (INT; Fig. 6*A*); (3) neurons modulated by INT and either the nine spatial positions (INT+POS; Fig. 6*A*) or (4) the epochs of interest (INT+EPO; Fig. 6*A*). Comparing the results in the three areas, we have found no statistical differences between the four categories of task-related cells in both V6A and PEc, whereas some dissimilarities appeared between the two visuomotor areas and PE: compared with PE, V6A and PEc contain a higher percentage of neurons modulated by eye position, arm position or both (POS+EPO, V6A 52%, PEc 48%, PE 28%; see Fig. 6*A*) and a higher incidence of neurons modulated by the interaction factor and target positions (INT+POS, V6A 16%, PEc 14%, PE 4%; see Fig. 6*A*; z-test, V6A vs PE, POS+EPO *p* = 1 × 10^−7^, INT+POS, *p* = 0.0001; PEc vs PE, POS+EPO *p* = 10^−5^, INT+POS, *p* = 0.0009; V6A vs PEc POS+EPO *p* = 0.3, INT+POS, *p* = 0.5). Also, the proportion of cells modulated by the interaction factor and epochs (INT+EPO) was similar in V6A and PEc (z-test, V6A vs PEc *p* = 0.1) with a lower incidence of these cells in V6A compared with PE (z-test, V6A vs PE, *p* = 0.01) and no significant difference between PEc and PE (z-test, PEc vs PE *p* = 0.2; V6A 4%, PEc 7%, PE 10%; see Fig. 6*A*). Cells modulated only by the interaction were similarly represented in all the three areas (V6A 3%, PEc 3%, PE 3%; see Fig. 6*A*; z-test, *p* > 0.05 for all comparisons).

### A reverse trend of eye-hand position tuning in V6A/PEc and PE

To quantify the proportion of cells tuned by the target positions in FIX, HOLD, and in both epochs, we performed a one-way ANOVA (*p* < 0.05). Figure 6*B* shows the incidence of task-related cells tuned by eye positions (FIX) and/or arm positions in space (HOLD) separately for each area. We found that the distribution of V6A, PEc, and PE cells based on their tuning for reaching targets was different across task epochs (Fig. 6*B*). Cells modulated only during FIX represented almost 30% of the total cell population in all three areas (z-test, *p* > 0.05 for all comparisons). Instead, cells modulated in HOLD and in both FIX and HOLD showed a clear and opposite trend from V6A to PE: cells modulated only by the position of the arm (HOLD) increased going from V6A to PE (z-test, V6A vs PEc, *p* = 4 × 10^−7^; V6A vs PE, *p* = 7 × 10^−8^), whereas cells modulated by eye-position and arm-position (FIX&HOLD) progressively decreased from V6A to PE (z-test, V6A vs PEc, *p* = 0.0008; V6A vs PE, *p* = 8 × 10^−9^; PEc vs PE, *p* = 0.002).

Moreover, within the same area we found some dissimilarities in the categories of cells spatially tuned in V6A and PEc, but not in PE. In V6A and PEc, cells spatially modulated during both FIX and HOLD were more represented (64% and 47% respectively) than those cells tuned only in one epoch (z-test, *p* < 0.01 for all comparisons), supporting the view that these areas are more implicated in eye-hand coordination, being highly sensitive to both the direction of gaze and to the arm proprioceptive signals. Furthermore, in V6A we observed a lower incidence of neurons modulated only during HOLD with respect to those modulated only during FIX (z-test, *p* = 3 × 10^−6^), and this is in line with the increase of somatosensory and the simultaneous decrease of visual processing observed along the caudo-rostral axis of the medial SPL. In PE we did not find any statistical differences in the subgroups of cells (z-test, *p* > 0.05 for all comparisons).

To sum up, these results show the existence of a decreasing trend for cells spatially modulated during both fixation and arm holding going from V6A to PEc and then to PE. This coding scheme parallels the gradual shift from joint to separate processing of amplitude and directions signals of arm movement during reaching (Hadjidimitrakis et al., 2014a, 2015; De Vitis et al., 2019).

### Dynamic space representation along the task

**Figure 7.**
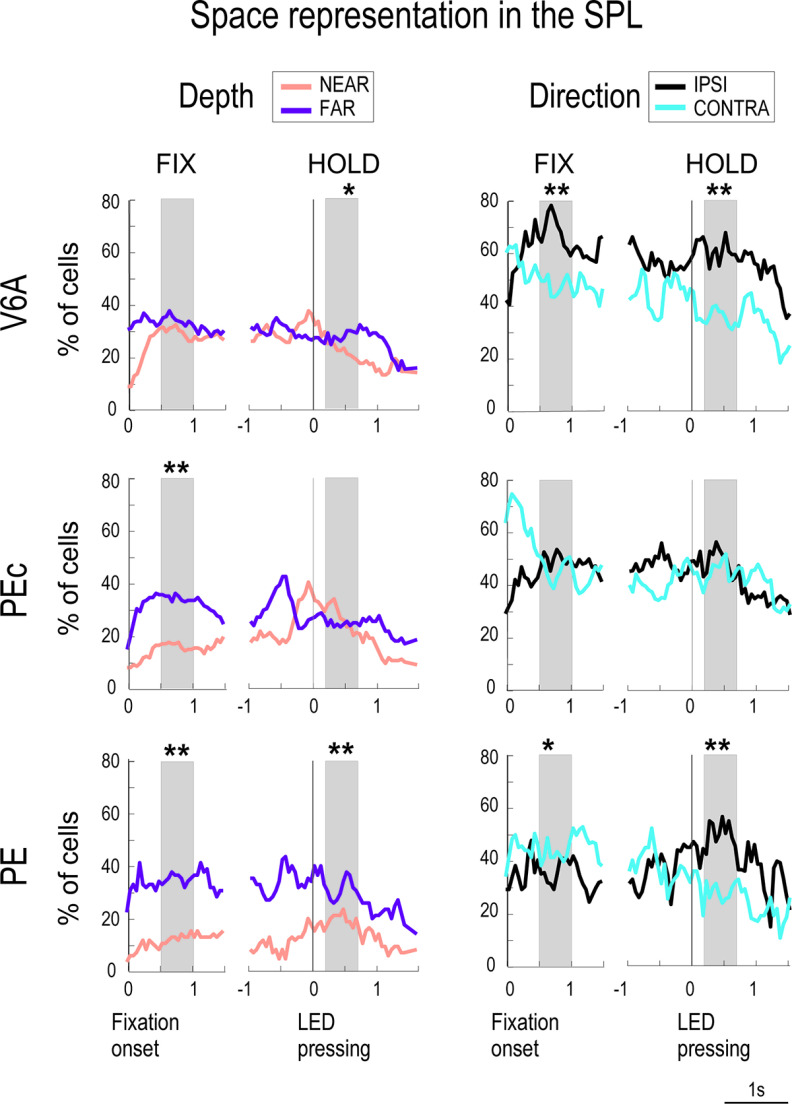
Space representation in the three SPL areas along the task. Percentage of V6A (top), PEc (middle), and PE (bottom) task-related cells linearly modulated by depth (left) and direction (right) showing a preference for far (FAR, purple line) or near (NEAR, pink line) space and ipsilateral (IPSI, black line) or contralateral (CONTRA, light blue line) space with respect to the recording hemisphere in a sliding window linear regression (window-bin width: 250 ms; step: 50 ms). Asterisks indicate significant differences between curves in bins of 250 ms (two-sample Kolmogorov–Smirnov test, *p* < 0.01). Other conventions as in Figure 3.

To characterize the spatial preference of task-related neurons during the time course of the task, a sliding window linear regression analysis was performed, considering target depth and direction as independent variables. Neurons with a significant linear vergence tuning were classified as NEAR or FAR, whereas cells linearly tuned by version angle were classified as CONTRA or IPSI, depending on both the sign of the correlation coefficient and the recording hemisphere. The percentage of V6A, PEc, and PE cells falling into the above groups is illustrated in Figure 7. Regarding the neuronal preference for depth, V6A neurons equally represented NEAR and FAR reachable space during the time course of the task, with a slight preference for farther positions at the end of the holding phase (Fig. 7, left, two-sample Kolmogorov–Smirnov test, *p* < 0.01). PEc cells showed instead a stronger tuning for FAR space from the beginning of the trial (FIX, two-sample Kolmogorov–Smirnov test, *p* < 0.01), and this predominance was maintained until movement execution, after which, in the HOLD phase, the proportion of neurons preferring FAR positions matched that of neurons preferring NEAR positions (Fig. 7, left, two-sample Kolmogorov–Smirnov test, *p* > 0.05). PE neurons tuned for FAR reachable space were found to be more represented than those tuned for NEAR space during the course of the whole trial (Fig. 7, left, two-sample Kolmogorov–Smirnov test, *p* < 0.01 in FIX and HOLD). This remarkable preference for FAR space likely reflects the strong influence of somatosensory input in PE. When the monkey reaches the farthest positions, the arm hyperextends to touch the target, and this leads to a strong somatosensory stimulation evoked by shoulder, elbow and wrist rotation. Regarding the directional tuning (Fig. 7, right), IPSI neurons were more numerous than CONTRA ones in V6A, particularly during target fixation and holding (two-sample Kolmogorov–Smirnov test, *p* < 0.01), whereas these two categories of neurons were equally represented in PEc (two-sample Kolmogorov–Smirnov test, *p* > 0.05 in FIX and HOLD). In turn, PE cells showed a gradual shift from a slight preference for the CONTRA space in the early part of the trial (two-sample Kolmogorov–Smirnov test, *p* < 0.05 in FIX) to a more pronounced preference for the IPSI space in HOLD (Fig. 7, right, two-sample Kolmogorov–Smirnov test, *p* < 0.01).

We then investigated how constant the preference for a given position (i.e., NEAR vs FAR or IPSI vs CONTRA) during the time course of the task was. To evaluate the consistency of spatial preference across single neurons, we quantified the cells that retained, altered, lost, or acquired their spatial preference in couples of subsequent bins of 50 ms. The overall tendency of cells from all the three areas was to retain their spatial preference both in depth and direction (i.e., vergence and version). Cells that did not alter their preference as the task progressed were the most represented in all the three areas (50–60% in V6A, 40–50% in PEc, 30–40% in PE), from fixation onset until target pressing, both in depth and direction. The proportion of cells preserving their depth tuning slightly decreased during target holding (epoch HOLD) in all three areas (40% in V6A, 30–40% in PEc, 20–30% in PE). Only a minority of cells from all the three areas (10–15%) lost or acquired their tuning, and very few cells (<3%) changed their spatial preference during the time course of the trial, both in depth and direction. In summary, the trends were similar for all the three areas both in depth and direction, with a remarkably stable proportion of spatially tuned cells that retained their tuning as the trial progressed, from target fixation to LED pressing.

### Population responses

**Figure 8.**
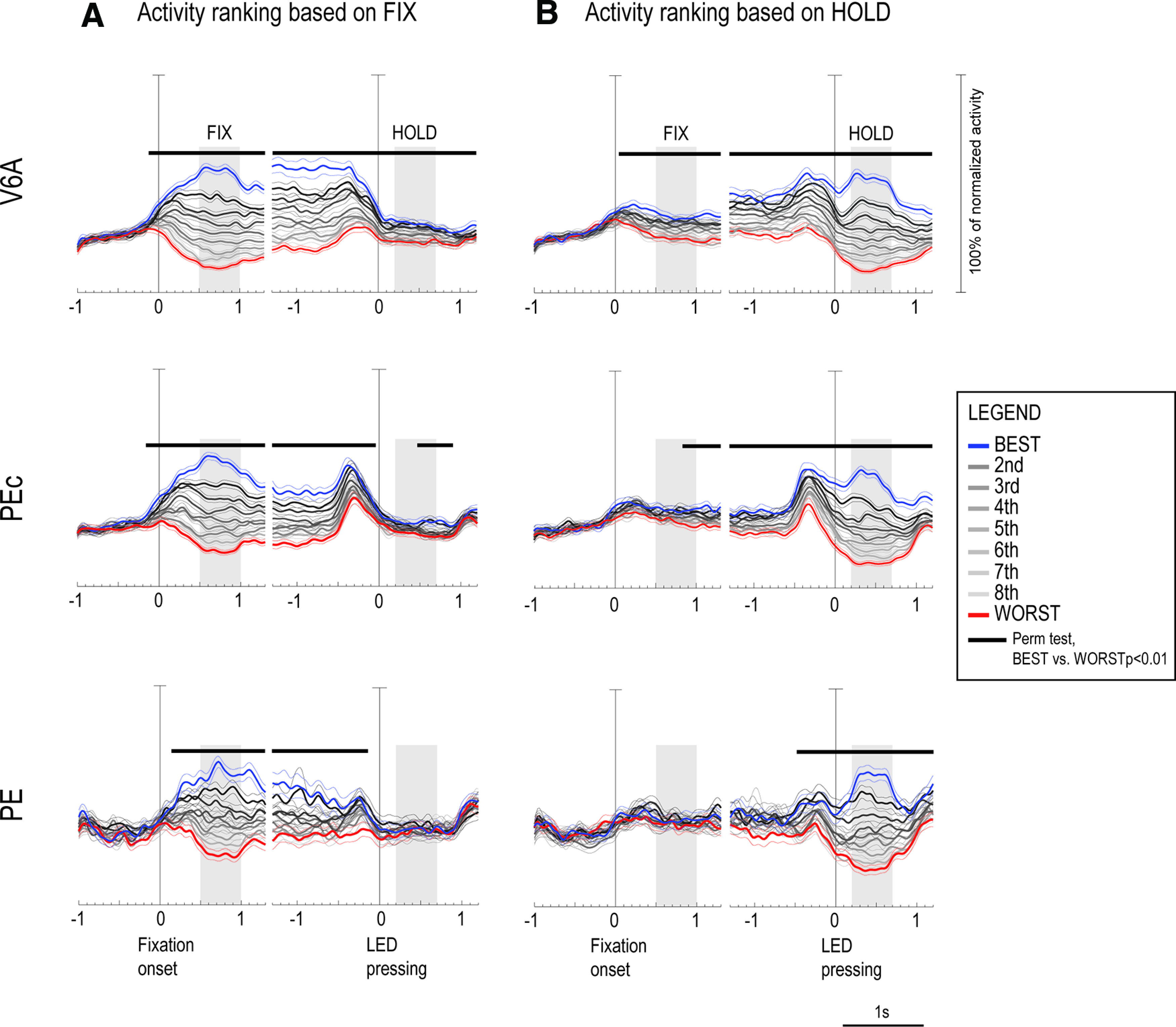
Population activity. Population activity of V6A (top), PEc (middle), and PE (bottom) cells modulated by the position of reaching target during FIX and/or HOLD, expressed as averaged normalized SDFs (thick lines) with variability bands (SEM; thin lines). ***A***, SDFs obtained by ranking the activity of each neuron according to the intensity of the response for each spatial position elicited in FIX for that neuron. ***B***, SDFs obtained ranking the activity of each neuron according to the intensity of the response for each spatial position elicited in HOLD. Neuronal activities have been aligned twice at the onset of fixation and at LED pressing. Vertical bars in all SDF plots: 100% of normalized activity. Permutation test was performed on BEST (blue line) and WORST (red line) curves in all the time intervals shown. At the top of each panel, black bars are used to indicate the significancy (see legend).

The timing of neural activity during the task and the spatial tuning of cells modulated by eye-position and/or arm-position is evident from the population SDFs (see Materials and Methods) shown in Figure 8, where neural activity was ranked according to each cell’s preference in FIX (Fig. 8*A*) and HOLD (Fig. 8*B*). In Figure 8*A*-*B*, cell responses were classified from the strongest one, elicited by a certain target position for a given neuron (whatever the position of the target was), hereafter called BEST, the second best response, the third, the fourth, and so on, up to the ninth, corresponding to the weakest response for the same neuron, hereafter called WORST, during FIX and HOLD epochs, respectively.

After ranking the neural activities according to the spatial preferences during FIX, we found that the activity during fixation started to diverge around the fixation onset in V6A and PEc (Fig. 8*A*, top and middle panels, permutation test, best vs worst curves, V6A *p* = 0.003, PEc *p* = 0.008), while in PE neural responses diverged later on, after the fixation onset (Fig. 8*A*, bottom panels, permutation test, PE *p* = 0.004). The size of the tuning was similar in the three areas in FIX, but the modulation lasted longer in V6A and PEc than in PE (compare the significance bars reported in each panel).

After ranking the neural activities according to spatial preferences during HOLD, we still found a tuning effect during FIX in V6A (Fig. 8*B*, top panels), where BEST and WORST position lines started to diverge 40 ms after the fixation onset and remained well separated for the remaining part of the trial (permutation test, best vs worst curves, V6A *p* = 0.007). During HOLD, the activity in V6A was strongly tuned: the curves for all nine conditions appeared to be unraveled and well distinct, with activities for the BEST and second best conditions being continuously higher than the baseline (FIX activity), and the ones for conditions seventh, eighth, and WORST being progressively more inhibited than the activity during FIX. In PEc (Fig. 8*B*, middle panels), the modulation during FIX, although significant (permutation test, best vs worst curves, PEc *p* = 0.008), was much weaker than in V6A, and in PE (Fig. 8*B*, bottom panels) it was completely absent (*p* > 0.05). During HOLD, the same strong tuning was present in PEc and PE, and it looked similar in the three areas. It is also evident, though not indicated by a specific epoch in Figure 8, that the activity during the execution of arm movement (the period just before the alignment to the LED pressing) was strongly tuned in V6A and PEc and weaker in PE.

Interestingly, while in V6A and, although to a lesser extent, PEc, the spatial tuning was evident during both FIX and HOLD, regardless of the epoch on which the ranking was based (Fig. 8*A* and 8*B*), in PE the modulations were evident only if the ranking was made according to the given epoch (i.e., clear tuning in FIX and no modulation in HOLD if the ranking was based on the activity in FIX, Fig. 8*A*; the opposite trend if the ranking was based on the activity in HOLD, Fig. 8*B*). This suggests that the ranking orders in PE were different during fixation and arm holding, and that the neural representations of these two signals (eye position, proprioception) in PE are independent. To sum up, V6A and PEc neurons showed similar temporal evolution in both FIX and HOLD, being their activity jointly influenced by eye position and arm movement-related information, whereas PE cells seemed to be more involved in the encoding of proprioceptive signals from the arm rather than oculomotor signals.

As evident from the population data, the activity for the WORST position in HOLD is lower than in FIX. This suggests that cells could be further inhibited by the position of the arm, in addition to the inhibition because of eye position. So, we calculated the incidence of task-related cells excited (i.e., with a higher firing rate) or inhibited (i.e., with a lower firing rate) during HOLD with respect to the baseline activity (FIX) in each target position. As expected, we found more cells inhibited than cells excited in all the three SPL areas we tested: 33 cells inhibited (15%) and 9 excited (4%) in V6A (z-test, V6A *p* = 0.0001), 25 cells inhibited (13%) and 10 excited (5%) in PEc (z-test, PEc *p* = 0.01) and 12 cells inhibited (14%) and 4 excited (5%) in PE (z-test, PE *p* = 0.04). The incidence of inhibition was similar among the three areas.

## Discussion

In this study, we investigated the modulating effect of eye-position and arm-position on neuronal activity in areas V6A, PEc, and PE while the animals performed a foveal reaching task. It has been recently suggested that V6A and PEc belong to the same cytoarchitectural sector of the SPL (Brodmann’s area 7), whereas PE does not (Brodmann’s area 5; Gamberini et al., 2020). Present data support this view showing similarities between the functional properties of V6A and PEc and differences with respect to PE. We have found that the incidence of task-related cells was lower in PE than in V6A and PEc (75% in V6A; 71% in PEc; 45% in PE) and showed that V6A and PEc neurons exhibited similar spatial patterns of neural modulation during fixation and target holding, according to the target position. In contrast, in PE the neural representations during fixation and target holding were not joined, suggesting an independent encoding of eye and arm position. A similar trend has been observed in the neural processing of amplitude and direction of arm movement during reaching: in PE the neural substrates related to amplitude and direction were different (Lacquaniti et al., 1995; De Vitis et al., 2019), while in V6A a common neural substrate was observed (Hadjidimitrakis et al., 2014a). Our results revealed a decreasing trend for cells spatially modulated during both fixation and arm holding going from V6A to PEc, to PE (Fig. 6*B*). This suggests that V6A and PEc may act as a bridge between pure visual cortices of the occipital lobe and rostral areas of the SPL more specifically involved in the proprioceptive control of action, like PE. This result is in line with the high sensitivity of both V6A and PEc to the direction of gaze, which is often a relevant cue to encode spatial coordinates of reaching targets (Galletti et al., 1995; Battaglia-Mayer et al., 2001; Ferraina et al., 2001; Raffi et al., 2008; Breveglieri et al., 2012; Hadjidimitrakis et al., 2014b), and to proprioceptive signals, particularly from the limbs (Breveglieri et al., 2002, 2008; Gamberini et al., 2018). The higher proportion of cells activated by a mix of gaze and arm signals in V6A and PEc than in PE (Fig. 6*B*) supports the view that these areas are more implicated in eye-hand coordination (Battaglia-Mayer et al., 2001; Ferraina et al., 2001; Diomedi et al., 2020).

The second trend emerging from our data is related to the incidence of cells modulated only during HOLD, which is much lower in V6A compared with PEc and PE. This result is consistent with the increasing percentage of neurons sensitive to somatosensory signals passing from V6A (∼30%; Breveglieri et al., 2002), to PEc (∼65%; Breveglieri et al., 2006, 2008; Gamberini et al., 2018), to PE (>90%; Sakata et al., 1973; Mountcastle et al., 1975; De Vitis et al., 2019). In line with this view, it has been reported a predominance of eye-centered and mixed (eye/hand-centered) cells in V6A (Bosco et al., 2016), and a prevalent employment of hand-centered reference frame in PEc (Hadjidimitrakis et al., 2014b; Piserchia et al., 2017) and PE (Lacquaniti et al., 1995).

### Role of PE in encoding arm posture

While in the motor cortex the maintenance of a steady position of the arm is more related to the patterns of muscular contraction rather than to the posture per se (Evarts, 1969; Cheney and Fetz, 1980; Fromm, 1983), the sensitivity of the majority of Brodmann’s area 5 cells to passive movements of the limbs (Sakata et al., 1973; Mountcastle et al., 1975) and to static arm positions (Georgopoulos et al., 1984; Kalaska and Hyde, 1985; Hamel-Pâquet et al., 2006; Cui and Andersen, 2011; McGuire and Sabes, 2011; Shi et al., 2013; De Vitis et al., 2019) has suggested a major role of this parietal area in encoding arm posture, pursuant to the current results regarding area PE. Despite the impact of the above cited papers about PE functional properties, only a few studies addressed the relative contribution of gaze direction and static hand positions signals in PE (Ferraina et al., 2009; De Vitis et al., 2019). Ferraina and colleagues’ results highlighted the effect of both eye and hand information on PE neuronal activity, with a prevalence of hand information. Conversely, our results suggest a similar encoding of eye and arm signals in PE. The discrepancy could be because of the different experimental conditions, being the task used by Ferraina and colleagues a non-foveated reaching task, where arm-target positions changed while the coordinates of fixation-target remained constant. Another explanation could be the difference in the recording sites, since they studied a lateral sector of area PE, that only partially overlapped with our recording region (compare the yellow area in Fig. 1 with Fig. 1*b* of De Vitis et al., 2019). Moreover, the data shown in Figures 6*B*, 8 suggest that an independent encoding of ocular and hand signals occurs in PE. This is in agreement with the independent encoding of version and vergence signals operated by PE neurons found by Lacquaniti (1995) and suggests that PE performs more specialized analysis of sensory signals than the regions located more caudally in the SPL, like PEc and V6A. Furthermore, we have found that a large proportion of PE cells tuned by depth showed a bias for FAR reachable space (Fig. 7), which most likely reflects a movement amplitude, corroborating the view that PE is involved in processing somatosensory and proprioceptive signals from the arm. Postural adjustments could be more important when the monkey reaches and holds the farthest targets and the integration of somatosensory and proprioceptive inputs from the arm could be reflected in increased levels of neural activity.

### Possible influence of spatial attention shifts on V6A activity

Given the foveal nature of the reaching movements in our task, we cannot exclude that the responses observed during static arm positions could also reflect the overt spatial attention directed to the target besides the gaze (eye position) and the proprioceptive cues (arm position). In addition, covert shifts of spatial attention, during which the attentional focus is decoupled from gaze, which allows to direct the attention to a peripheral location without moving the eyes, may have contributed to modulate V6A neural activity. Several monkey and human experiments have revealed a crucial role of SPL during both overt and covert spatial attention shifts (Vandenberghe et al., 2001; Yantis et al., 2002; Molenberghs et al., 2007; Kelley et al., 2008; Galletti et al., 2010; Ciavarro et al., 2013; Caspari et al., 2015, 2018; Arsenault et al., 2018). In 2010, Galletti and colleagues showed for the first time attention-related activity in V6A at single cell level using a task where the monkey was required to covertly shift its attention from a central fixation point toward a peripheral location, and vice versa (Galletti et al., 2010). They found that the neural modulation was still present when attention was covertly shifted outward, to a peripheral cue, and demonstrated that visual, motor, and attentional responses can occur in combination in single V6A neurons. More recently, Caspari and colleagues (Caspari et al., 2015) have identified in monkeys a network of areas, including parietal area V6A, activated during spatial shifting events, using a spatial attention task adapted from a human fMRI study (Molenberghs et al., 2007). These findings could explain the proportion of V6A cells that we found to be inhibited during HOLD, possibly because of the modulating effect of the spotlight of attention. During HOLD, the spatial attention is likely to be covertly shifted out of the reaching target because the animal at that time has a more attractive object to attend -the HB that must be reached by its hand soon after the HOLD period to receive a reward.

We observed a similar percentage of cells inhibited during the target holding in all the SPL areas studied (15% in V6A, 13% in PEc, 14% in PE). Inhibited cells were also found by Gardner et al. (2007) in PE (41%) and AIP (38%) during the holding period after a grasping movement in a reach-to-grasp task. The inhibition was particularly relevant near the end of the holding period, just before the start of the backward arm movement to reach the initial hand position. Even in this case, spatial attention shifts (from positions on the panel, the reaching targets, to a position near the trunk, the HB) could explain the observed results.

### SPL lesions impair visuomotor coordination during reaching

The SPL sectors studied here are often damaged in patients affected by optic ataxia, a visuomotor coordination deficit that strongly impairs reaching actions (Rossetti et al., 2019). Very recent studies on optic ataxia patients performing reaching actions relying exclusively on proprioception showed that SPL lesions cause larger position errors than in healthy controls (Bartolo et al., 2018; Mikula et al., 2021). These studies highlight the crucial role of these regions in using proprioceptive information about hand position to correctly direct reaching movements (Mikula et al., 2021). Present data on single cells recording from monkey SPL may be the neurophysiological counterpart of this finding, with a deeper understanding of the stronger role of PE in estimating hand position basing on proprioceptive information and of V6A and PEc in linking this input with gaze-related signals as well as visual inputs. All these areas are well equipped to contribute to the state estimation about upper limb status for controlling the correct execution of reaching movements (Fattori et al., 2017).

In conclusion, present data show that eye-position and arm-position modulation of neuronal activity is similar in areas V6A and PEc, and different in area PE. These results agree well with the recent suggestion that both V6A and PEc belong to Brodmann’s area 7 while PE to Brodmann’s area 5 (Gamberini et al., 2020). According to this view, we found that all three SPL areas integrate eye and limb position signals during the hand holding at the end of a foveal reaching, but the influence of the two signals is different in areas V6A and PEc with respect to PE. Area PE was found to be more sensitive to limb proprioceptive input while PEc and V6A, particularly this latter, were also influenced by the direction of gaze. These data support the existence, often reported in literature (Piserchia et al., 2017; Gamberini et al., 2018; De Vitis et al., 2019; Impieri et al., 2019), of a functional trend in the SPL, with the anterior part more involved in limb representation and the posterior one showing visuomotor characteristics well suited to control goal-directed actions.

## Conclusion

Present data show that eye-position and arm-position modulation of neuronal activity is similar in areas V6A and PEc, and different in area PE. These results agree well with the recent suggestion that both V6A and PEc belong to Brodmann’s area 7 while PE to Brodmann’s area 5 (Gamberini et al., 2020). According to this view, we found that all three SPL areas integrate eye and limb position signals during the hand holding at the end of a foveal reaching, but the influence of the two signals is different in areas V6A and PEc with respect to PE. Area PE was found to be more sensitive to limb proprioceptive input while PEc and V6A, particularly this latter, were also influenced by the direction of gaze. These data support the existence, often reported in literature (Piserchia et al., 2017; Gamberini et al., 2018; De Vitis et al., 2019; IImpieri et al., 2019), of a functional trend in the SPL, with the anterior part more involved in limb representation and the posterior one showing visuomotor characteristics well suited to control goal-directed actions.

## Synthesis

Reviewing Editor: Satu Palva, University of Helsinki Decisions are customarily a result of the Reviewing Editor and the peer reviewers coming together and discussing their recommendations until a consensus is reached. When revisions are invited, a fact-based synthesis statement explaining their decision and outlining what is needed to prepare a revision will be listed below. The following reviewer(s) agreed to reveal their identity: NONE.

This is an interesting study reporting the effects of gaze and arm position in the primary superior parietal lobule. The authors have trained monkeys to perform a challenging reaching task under conditions of varying gaze and obtained a large dataset of neurons in areas V6A, PEc, and PE. They compare the properties of neurons in each. The experiments are well conducted and the results clear and straightforward. Presentation of the results and conclusions could be improved still.

A. The motivation behind many of the experiments and analyses is unclear, and the significance of the findings is not made explicit. The “Significance Statement” at the very beginning of the paper exemplifies the problem. This section mostly restates the results. The paragraph should be rewritten. The last page of the introduction does a better job in that respect.

B. Presentation of the results is very terse. The text reads like a series of facts and numbers with little context. Are there competing hypothesis that the authors wish to adjudicate between? What are the implications between different potential outcomes of each experiment and analysis? Only in the discussion does the reader become to grasp the motivation behind the analyses conducted.

C. Label the Y-axis in Figure 7 (even if to show the normalized scale).

D. Page 12, line 17: What did the permutation test compare?

E. Breaking the discussion in sections would improve readability and conveying the authors' message.

F. The results shown in figure 7 could benefit about some further aspects of the population encoding. What about the proportion of neurons preferring ipsi over contra reaches or holding positions? What about preferences of near vs far? I guess it would be interesting to see if space (ipsi vs contra, near vs far) is equally covered by neurons from all three areas. This could be analyzed as average over time but also concerning the dynamics. Do neurons anticipate their “preferred gaze/reach direction/depth”? Are dynamics similar or different across areas? Results might be interesting not only for experimentalists, but also theoreticians.

G. The manuscript would benefit from another round of editing. A (non-exhaustive) list of issues that I compiled as I went along the text:

1. Page 1, line 1: “fascicularis” should be spelled with a lower case “f"

2. Page 1, line 14: rephrase “Although with different features...” Unclear. Rephrase.

3. Page 7, line 20: use of the division “ ÷ “ sign is not recommended in equations. The “ / “ is clearer (and the authors use it repeatedly in the same paragraph).

4. Page 9, line 13: First complete sentence should read “A V6A neuron...”

5. Page 9, line 20: Beginning of sentence should read “In contrast to V6A...”

6. Page 10, line 17: Delete “If"

7. Page 14, line 21: “way lower” is colloquial. Rephrase.

8. Page 17, line 21: rephrase to “agree well”.
